# Adipose expression of CREB3L3 modulates body weight during obesity

**DOI:** 10.1038/s41598-021-98627-z

**Published:** 2021-09-29

**Authors:** Maximilian A. McCann, Yanliang Li, Marcos Muñoz, Victoria Gil, Guifen Qiang, Jose Cordoba-Chacon, Matthias Blüher, Stephen Duncan, Chong Wee Liew

**Affiliations:** 1 Department of Physiology & Biophysics, University of Illinois at Chicago, Chicago, IL USA; 2 Department of Endocrinology, Huashan Hospital, Fudan University, Shanghai, China; 3 Department of Medicine, Section of Endocrinology, Diabetes and Metabolism, University of Illinois at Chicago, Chicago, IL USA; 4 Helmholtz Institute for Metabolic, Obesity and Vascular Research (HI-MAG) of the Helmholtz Zentrum München at the University of Leipzig and University Hospital Leipzig, Leipzig, Germany; 5 Department of Regenerative Medicine & Cell Biology, Medical University of South Carolina, Charleston, SC USA; 6 Department of Physiology & Biophysics, College of Medicine, University of Illinois at Chicago, 835 S Wolcott Ave, M/C 901, MSB, E-202, Chicago, IL 60612 USA; 7 Present Address: State Key Laboratory of Bioactive Substances and Functions of Natural Medicines, Institute of Materia Medica, Chinese Academy of Medical Sciences and Peking Union Medical College, Beijing, China

**Keywords:** Fat metabolism, Homeostasis, Metabolic diseases

## Abstract

We found the hepatic transcription factor Cyclic-AMP Responsive Element Binding Protein 3-like-3 (CREB3L3) to be expressed in adipose tissue, and selectively downregulated in the more metabolically protective subcutaneous adipose tissue in obese mice and humans. We sought to elucidate the specific role of this factor in adipose biology. CREB3L3 fat-specific knockout mice were fed a high-fat diet to induce obesity and metabolic dysfunction. Additionally, we injected a flip-excision adeno-associated virus directly into the subcutaneous inguinal adipose tissue of Adiponectin-Cre mice to create a depot-specific overexpression model for further assessment. Fat-specific ablation of CREB3L3 enhanced weight gain and insulin resistance following high-fat feeding, as fat-specific knockout mice expended less energy and possessed more inflammatory adipose tissue. Conversely, inguinal fat CREB3L3 overexpression deterred diet-induced obesity and ameliorated metabolic dysfunction. Together, this study highlights the relevance of CREB3L3 in obese adipose tissue and demonstrates its role as a powerful body weight modulator.

## Introduction

Obesity is the result of an energy imbalance where caloric intake exceeds energy expenditure, causing extra calories to be stored within adipose tissues throughout the body. The expansion of adipose tissues during obesity leads to several changes in the biology of the tissue. Obese adipose tissue undergoes changes in lipid metabolism, inflammation, and adipokine secretion. This leads to increased release of free fatty acids and pro-inflammatory cytokines, in addition to shifting the secretion of adipokines. These shifts in the adipose secretome drive lipotoxicity, inflammation, and eventual insulin resistance in other metabolic organs, culminating in the development of metabolic syndrome^1–5^.

The impact of these changes in adipose biology differ between fat depots during obesity. Among the white adipose tissues, visceral fat enlargement strongly correlates with the development of metabolic syndrome^6^. This is believed to be due to the more inflammatory nature of this tissue^7^ and its enhanced ability to mobilize fatty acids via lipolysis^8^. Conversely, the subcutaneous fat is seen as being more “metabolically protective” during obesity and individuals with more subcutaneous fat deposition are less likely to develop metabolic syndrome^9,10^. Subcutaneous fat is believed to be more protective because it is more plastic, secretes more beneficial hormones, and is more metabolically flexible than visceral fat^11^. Subcutaneous fat is able to form brown-like “beige” adipocytes in response to cold or beta adrenergic stimulation^12^, whereas murine visceral fat is virtually incapable of undergoing browning, except in select models^13–15^. While the physiological basis for the differential effects that visceral and subcutaneous fat have during obesity are known, the cellular mechanisms behind these divergent metabolic profiles have not been fully characterized.

Cyclic-AMP Responsive Element Binding Protein 3-like-3 (CREB3L3) is an endoplasmic reticulum (ER)-bound transcription factor, which features a transmembrane domain that tethers it to the ER membrane^16^. CREB3L3 is activated by various stimuli in the liver including ER stress, pro-inflammatory cytokines^17^, fatty acids, insulin^18^, and the circadian oscillator BMAL1^19^. In response to these stimuli, CREB3L3 is trafficked to the Golgi, where it is cleaved by site-1 and site-2 proteases. This frees the transcription activation domain, allowing it to translocate to the nucleus and induce transcription^17^. Previous works have shown that CREB3L3 promotes the transcription of genes involved in gluconeogenesis^20^ and the acute phase response^17^ in the liver, in addition to being an important regulator of lipid metabolism^21^. CREB3L3 has been shown to regulate several aspects of lipid metabolism in the liver by inducing expression of apolipoproteins^22,23^, fibroblast growth factor 21 (Fgf21)^24^, genes involved in fatty acid oxidation^18^, and suppresses de novo lipogenesis through inhibition of sterol regulatory element-binding transcription factor 1c (SREBP1c)^25^.

Previous works have all described the function of CREB3L3 within the liver, and it has been deemed hepatic CREB (CREBH) in the literature. CREB3L3 has been described as a liver-specific factor^17,26^, with reports of similar expression levels in the ileum and lower expression levels in the pyloric stomach and duodenum as well^27^. In this paper, we report that CREB3L3 is not only expressed in adipose tissue, but selectively downregulated in subcutaneous fat in both obese humans and mouse models. Adipose-specific ablation of this transcription factor led to an enhancement of diet-induced obesity and insulin resistance in mice via a suppression of energy expenditure and increased adipose inflammation. Overexpression in the subcutaneous fat afforded mice protection from diet-induced obesity. Our results demonstrate the key role that CREB3L3 plays in modulating body weight and preventing pathological changes in both subcutaneous and visceral fat during obesity.

## Results

### CREB3L3 is expressed in adipose tissue and selectively downregulated in obese subcutaneous fat

**Figure 1:**
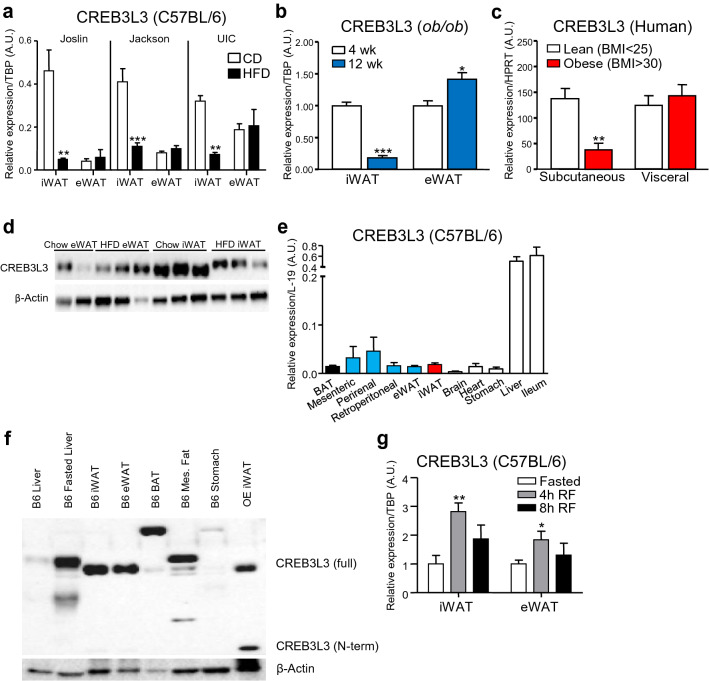
CREB3L3 is expressed in adipose tissue and selectively downregulated in obese subcutaneous fat. (**a**, **b**) Quantitative PCR for CREB3L3 message in inguinal (iWAT) and epididymal (eWAT) white adipose tissue from (**a**) C57BL/6 mice fed a high-fat diet for 12 weeks at the indicated animal facility (n = 6 mice per group) or (**b**) *ob/ob* mice before (4 week) and after (12 week) developing obesity (n = 6 mice per group). CREB3L3 expression was normalized to TBP housekeeping gene and presented as (**a**) relative expression or (**b**) fold change over lean controls. (**c**) Quantitative PCR for CREB3L3 message in subcutaneous abdominal and omental fat from patients with a BMI ≤ 25 kg/m^2^ (lean, n = 69) and BMI > 30 kg/m^2^ (obese, n = 504). CREB3L3 expression was normalized to HPRT housekeeping gene. (**d**) Western blot measuring abundance of CREB3L3 in iWAT and eWAT from adult C57BL/6 mice fed chow or high-fat (HFD) diet for 12 weeks. (**e**) Quantitative PCR for CREB3L3 message in various tissues from lean C57BL/6 mice (n = 3 mice). CREB3L3 expression was normalized to L-19 housekeeping gene. (**f**) Western blot measuring abundance of CREB3L3 in various tissues from same lean C57BL/6 mouse, fasted C57BL/6 liver, or iWAT with overexpression of CREB3L3. (**g**) Quantitative PCR for CREB3L3 message in iWAT and eWAT from adult C57BL/6 mice that were fasted for 24 h or subsequently refed (RF) for 4 or 8 h (n = 4–5 mice per group). CREB3L3 expression was normalized to TBP housekeeping gene and presented as fold change over fasting group. Data are presented as mean ± SEM with sample sizes listed above. The difference in means was analyzed using Student’s t-test where *P < 0.05, **P < 0.01, and ***P < 0.001.

In previous reports, our lab has identified multiple proteins that serve different functions in subcutaneous inguinal (iWAT) and visceral epididymal white adipose tissue (eWAT)^15,28,29^. As part of the data mining process for these studies, we identified the ER-bound transcription factor cyclic-AMP Responsive Element Binding Protein 3-like-3 (CREB3L3) as a protein that is differentially regulated in obese iWAT and eWAT. This phenomenon was observed in different cohorts of C57BL/6J mice that were fed a high-fat diet (HFD) for 12 weeks (Fig. 1a). The mice at UIC and the Joslin Diabetes Center had been bred for several generations, meaning their microbiomes differed from the mice housed at Jackson Labs. We therefore conclude that microbiome, environment, and genetic drift have no influence on obesity-induced changes in adipose CREB3L3 expression that we observed. Similarly, this specific downregulation of CREB3L3 was observed in the iWAT of a genetic model of obesity (*ob/ob*) (Fig. 1b), and was also seen at the protein level, as evidenced by the reduced abundance of CREB3L3 in the iWAT of C57BL/6J mice following high-fat feeding (Fig. 1d).

A cohort of obese patients (BMI > 30) were found to have a significant reduction in the CREB3L3 message present in their abdominal subcutaneous, but not omental fat, compared to lean patients (BMI < 25) (Fig. 1c). Additionally, CREB3L3 expression in patient subcutaneous fat negatively correlated with body fat mass, while visceral fat expression was positively correlated (Table 1). To determine if adipose CREB3L3 expression is associated with obesity-associated metabolic dysfunction, the correlation with glycated hemoglobin A1c (HbA1c) and fasting plasma glucose and insulin were examined. While HbA1c was significantly associated with CREB3L3 expression in both fat depots, there were no associations with fasting plasma glucose. Given that HbA1c levels are multifactorial and likely regulated by multiple organs, the effect of adipose CREB3L3 on HbA1c is likely indirect.

**Table 1:** Univariate correlation analyses (Pearson) of CREB3L3 human adipose tissue gene expression and metabolic traits.

Parameter	Visceral AT CREB3L3		SC AT CREB3L3	
	r	p	r	p
BMI	0.06	0.52	− 0.013	0.71
Body fat mass (%)	0.16	0.008	− 0.222	0.002
Fasting plasma glucose (mmol/L)	− 0.013	0.82	0.034	0.44
HbA1c (%)	0.147	0.02	0.279	< 0.001
Fasting plasma insulin (pmol/L)	0.05	0.24	− 0.084	0.18

Since this transcription factor had been previously described as being specifically expressed in the liver and ileum^27^, quantitative real-time PCR (qPCR) was performed using cDNA from various tissues from lean C57BL/6J mice to confirm the presence of CREB3L3 message within the adipose tissues under normal physiologic conditions (Fig. 1e). CREB3L3 message was in high abundance in the liver and ileum, but was also detected in several adipose tissues. CREB3L3 message was present in the brown adipose tissue (BAT), iWAT, as well as the visceral eWAT, mesenteric, and retroperitoneal depots. The expression in adipose tissues was at levels similar to those observed in the stomach, which has been previously shown to express CREB3L3^27^. Western blotting was performed to confirm these findings at the protein level. CREB3L3 protein was detected in the iWAT, eWAT, BAT, and mesenteric fat, while lower abundance of the protein was found in the liver and stomach from the same mouse (Fig. 1f). This represents the first reported CREB3L3 protein abundance data in various tissues and the relatively high expression of CREB3L3 in the liver did not translate to higher protein abundance. Since the abundance of protein in the liver does not match the higher mRNA expression, we included a fasting liver sample to demonstrate that the antibody could detect CREB3L3 in the liver. The abundance of CREB3L3 in the liver drops during the fed state^30^, whereas iWAT and eWAT CREB3L3 expression peaked after short-term refeeding (4 h) and is not different under fasted and long-term refeeding (8 h) conditions (Fig. 1g). Due to its higher abundance of CREB3L3, a fasting liver sample was used as a positive control, along with iWAT from mice with adipose overexpression of CREB3L3. Additionally, the protein in the liver, as well as the brown and mesenteric fat samples, is larger than in the iWAT and eWAT, which is closer to the expected protein size. This may be due to post-translational modifications that are known to occur in the liver^19,24,30^.

### Fat-specific ablation of CREB3L3 enhances diet-induced obesity and insulin resistance

**Figure 2:**
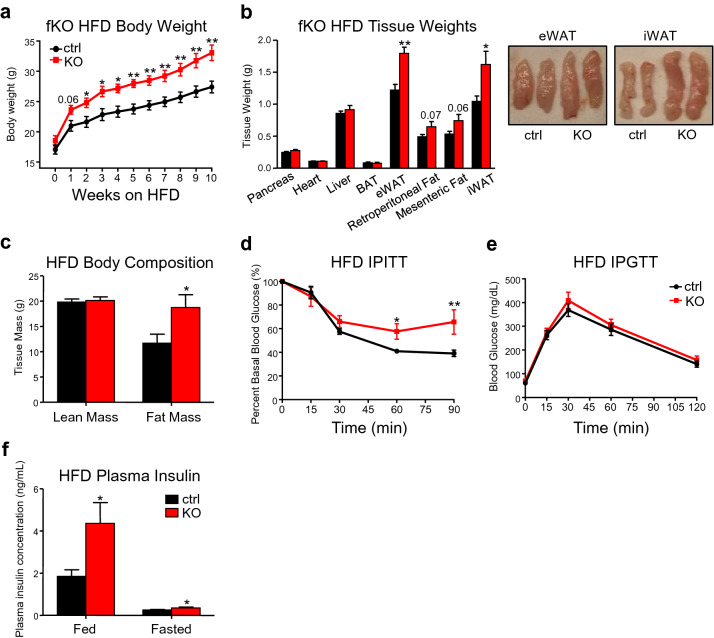
Fat-specific ablation of CREB3L3 enhances diet-induced obesity and insulin resistance. (**a**) Weekly body weight measurements of CREB3L3 floxed controls (ctrl) and fat-specific knockout (fKO) mice during 10 wks of high-fat diet feeding (n = 4–6 mice per group). (**b**) Tissue weight measurements for control and fKO mice after high-fat feeding (n = 4–6 mice per group). (**c**) Body composition following high-fat feeding measured by NMR (n = 6–10 mice per group). (**d**, **e**) Intraperitoneal (**d**) insulin and (**e**) glucose tolerance tests performed with high fat-fed control and fKO mice (n = 6–8 mice per group). (**f**) Fed and fasted plasma insulin concentrations measured with ELISA (n = 12–17 mice per fed group; 8 mice per fasted group). Data presented as mean ± SEM with sample sizes listed above. The difference in means was analyzed using Student’s t-test where *P < 0.05 and **P < 0.01.

To study the role of CREB3L3 in adipose biology, fat-specific knockout (fKO) mice were generated using Adiponectin-Cre mice^31,32^. The specific knockout of CREB3L3 was confirmed by separating mature adipocytes from iWAT and eWAT in lean fKO mice (Supplemental Fig. S1). While fKO did not alter body weight in chow-fed mice, (Supplemental Fig. S1), it increased NMR-based fat mass (Supplemental Fig. S1). Accordingly, when fKO mice were placed on HFD, they became significantly heavier than CREB3L3-floxed control littermates after two weeks of high-fat feeding (Fig. 2a). The changes in body weight are driven by significant enlargement of the iWAT and eWAT in the fKO mice (Fig. 2b, Supplemental Fig. S1). The retroperitoneal and mesenteric fat also trend towards being larger in the fKO mice. Together, the fKO mice had a 60 percent increase in fat mass, while there were no changes in the lean mass (Fig. 2c).

Coinciding with their larger fat mass, the fKO mice fed a HFD had reduced insulin sensitivity, as evidenced by the upward shift in the 60 and 90 min time points on the intraperitoneal insulin tolerance test (Fig. 2d), which was associated with increased levels of plasma insulin in both the fed and fasted states (Fig. 2f). Despite being more insulin resistant, fKO mice with diet-induced obesity show normal glucose clearance that suggests that beta cell function is not altered by fKO (Fig. 2e).

### CREB3L3 fKO promotes adipose inflammation and insulin resistance during obesity

**Figure 3:**
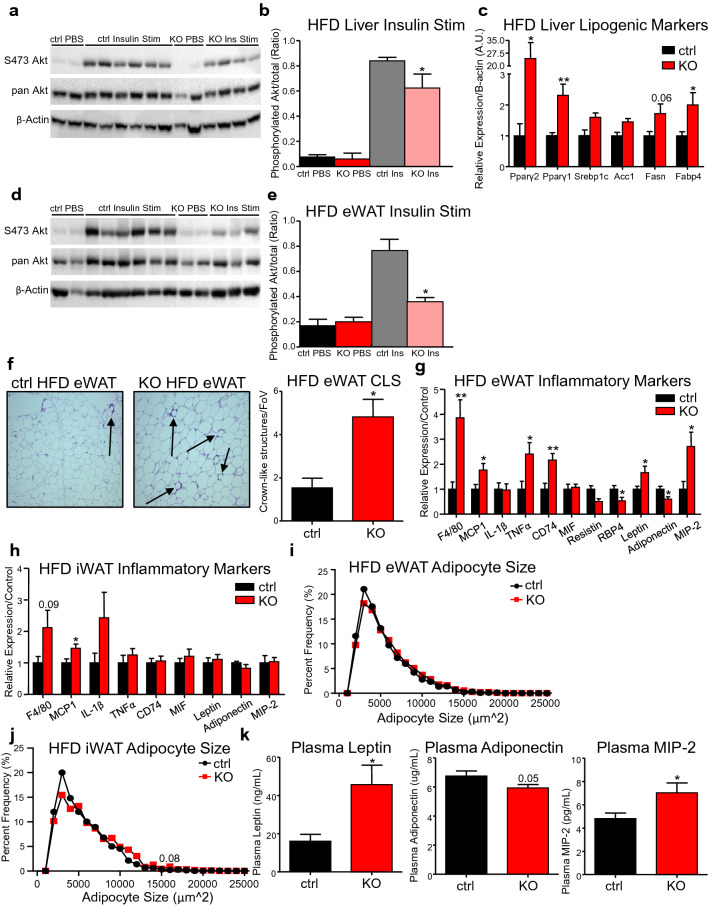
CREB3L3 fKO promotes insulin resistance and adipose inflammation during obesity. (**a**) Western blot measuring abundance of Akt and Akt phosphorylated at the S473 site in high fat-fed control and fKO livers following injection with PBS or insulin and (**b**) quantification of Western blot results, with abundance of S473 phosphorylation normalized to abundance of total Akt in liver (n = 2 mice per PBS group; n = 4–6 ins-stimulated mice per group). (**c**) Quantitative PCR for markers of lipogenesis in control and fKO livers following high-fat feeding (n = 5 mice per group). Lipogenic marker expression was normalized to B-actin housekeeping gene and presented as fold change over controls. (**d**) Western blot measuring abundance of Akt and Akt phosphorylated at the S473 site in high fat-fed control and fKO eWAT following injection with PBS or insulin and (**e**) quantification of Western blot results, with abundance of S473 phosphorylation normalized to abundance of total Akt in eWAT (n = 2 mice per PBS group; n = 3–6 insulin-stimulated mice per group). (**f**) Representative images of H&E-stained eWAT sections following high-fat feeding and quantification of the number of crown-like structures per field of view. Arrows demarcate the presence of crown-like structures (3 images were taken per mouse. n = 4–8 mice per group). (**g**) Quantitative PCR for markers of inflammation in control and fKO eWAT following high-fat feeding (n = 8–9 mice per group). Target genes were normalized to TBP expression and presented as fold change over controls. (**h**) Frequency distribution of the adipocyte areas of control and fKO eWAT following high-fat diet (10 images per mouse were used. n = 5 mice per group). (**i**) Quantitative PCR for markers of inflammation in control and fKO iWAT following high-fat feeding (n = 5–6 mice per group). Target genes were normalized to TBP expression and presented as fold change over controls. (**j**) Frequency distribution of the adipocyte areas of control and fKO iWAT following high-fat diet (10 images per mouse were used. n = 5 mice per group). (**k**) Quantification of plasma leptin and MIP-2 concentration following high-fat feeding using multiplex (n = 4–6 mice per group). Adiponectin concentration was measured using ELISA (n = 13–16 mice per group). Data presented as mean ± SEM with sample sizes listed above. The difference in means was analyzed using Student’s t-test where *P < 0.05 and **P < 0.01.

In order to determine tissue-specific insulin sensitivity, we assessed insulin-mediated phosphorylation of Akt. Interestingly, insulin-mediated phosphorylation of Akt at the S473 residue was significantly reduced in fKO livers following insulin stimulation (Fig. 3a,b) and the expression of insulin-like growth factor binding partner 1 was fourfold higher (Supplemental Fig. S2), indicative of hepatic insulin resistance^33^. Accordingly, the ability of insulin to promote de novo lipogenesis was increased as shown by the upregulation of SREBP1c, acetyl-CoA carboxylase (Acc1, also known as Acaca), fatty acid synthase (Fasn), and fatty acid binding protein 4 (Fabp4) (Fig. 3c)^34^. Despite the dramatic upregulation of hepatic peroxisome proliferator-activated receptor gamma (24-fold increase of Pparg2) (Fig. 3c), and its well-known steatogenic effects^35,36^; 10 weeks of HFD did not significantly alter the morphology (Supplemental Fig. S2) nor the triglyceride, cholesterol, or non-esterified fatty acid contents of these livers (Supplemental Fig. S2).

While the liver is a key contributor to insulin sensitivity^37^, we also assessed the contributions of adipose tissue to the insulin resistance observed in the fKO mice (Fig. 2d). Insulin-mediated phosphorylation of Akt was also reduced in the eWAT (Fig. 3d,e) and iWAT (Supplemental Fig. S3) following insulin stimulation. To decipher a potential cause of the insulin resistance in the fKO mice, we analyzed the morphology of the fKO adipose tissues with diet-induced obesity. Both the eWAT and iWAT from fKO mice had a significant increase in the number of crown-like structures (Fig. 3f, Supplemental Fig. S3), which are formations of macrophages around apoptotic adipocytes. This result was confirmed in the eWAT by fourfold higher expression of the macrophage marker F4/80 and upregulation of monocyte chemoattractant protein 1 (MCP1, also known as Ccl2) (Fig. 3g). While expression of MCP1 was significantly higher in fKO iWAT, the increased expression of F4/80 was more modest and not significantly different from the expression in control iWAT (Fig. 3h).

The increased macrophage infiltration in the fKO eWAT occurred without an increase in the frequency of hypertrophic adipocytes in the fKO eWAT (Fig. 3i), whereas the iWAT exhibited a shift towards having more hypertrophic adipocytes (Fig. 3j). While the fKO iWAT had increased expression of lipogenic genes like SREBP1c and Fasn (Supplemental Fig. S3), the tissue did exhibit reduced expression of the adipogenic transcription factor CCAAT enhancer binding protein alpha (C/EBPα) (Supplemental Fig. S3), which could contribute to the shift towards larger adipocytes observed in the fKO iWAT (Fig. 3j).

Along with the increased macrophage infiltration, there was a significant increase in the eWAT expression of the pro-inflammatory cytokine tumor necrosis factor alpha (TNFα), macrophage inflammatory protein 2 (MIP-2, also known as Cxcl2), and the macrophage migration inhibitory factor receptor CD74 (Fig. 3g). Additionally there was increased expression of leptin, which has pro-inflammatory effects^38^ and reduced expression of the insulin-sensitizing hormone adiponectin, which has anti-inflammatory effects^39^ (Fig. 3g). These changes in adipokine and cytokine expression were not observed in the fKO iWAT (Fig. 3h). Measuring the plasma concentration of these adipokines revealed that these expression changes in the eWAT translate to systemic differences in leptin and adiponectin abundance, in addition to higher concentrations of MIP-2 (Fig. 3k). Together, these results show that the adipose tissue from the fKO mice becomes more inflamed in the obese state, but the eWAT expresses more pro-inflammatory cytokines and adipokines, making it the more likely contributor to the insulin resistance observed in the liver and adipose tissue of the fKO mice.

### CREB3L3 fKO promotes weight gain by reducing energy expenditure and shifting brown fat lipid metabolism

**Figure 4:**
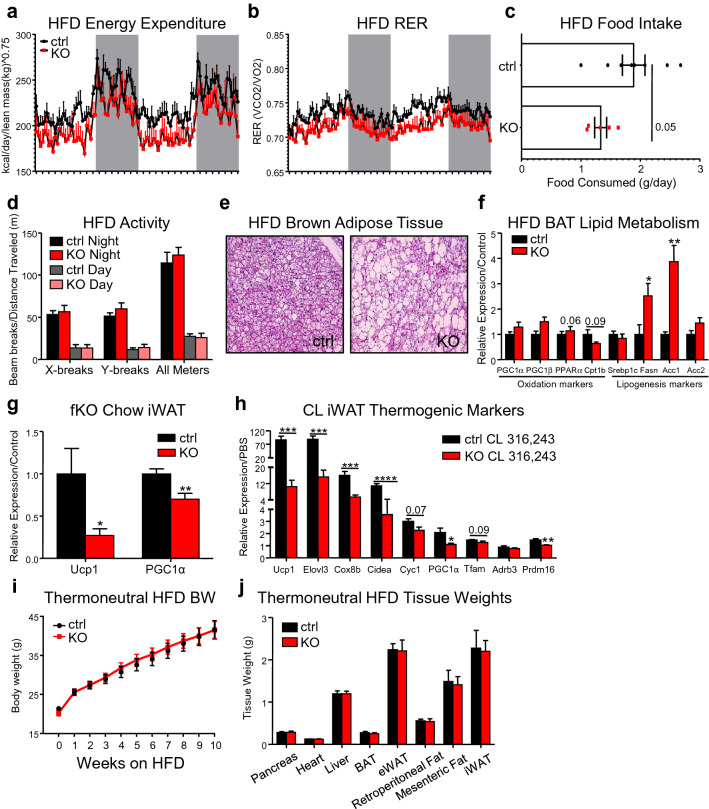
CREB3L3 fKO promotes weight gain by reducing energy expenditure and shifting brown fat lipid metabolism. (**a**) Energy expenditure and (**b**) respiratory exchange ratios (RER) of control and fKO mice fed high-fat diet (n = 6–8 mice per group). Energy expenditure was normalized to the animal’s lean mass. The curves for both measurements were analyzed using two-way ANOVA. P values for the column factor (genotype) for the energy expenditure and the RER were both < 0.0001. (**c**) Average daily consumption of high-fat diet while mice were in metabolic cages (n = 5–8 mice per group). (**d**) Activity measurements of control and fKO mice fed high-fat diet. Data presented as x- and y-axis beam breaks and total movement per day following acclimation period (n = 6–8 mice per group). (**e**) Representative images of H&E-stained brown adipose tissue sections following high-fat feeding. (**f**) Quantitative PCR for markers of fatty acid oxidation and lipogenesis in control and fKO brown adipose tissue following high-fat feeding (n = 5 mice per group). Expression was normalized to TBP and presented as fold change over controls. (**g**) Quantitative PCR for UCP1 and PGC1a in control and fKO inguinal adipose tissue in mice fed a chow diet (n = 5–6 mice per group). Target gene expression was normalized to TBP and presented as fold change over controls. (**h**) Quantitative PCR for markers of thermogenesis and adipocyte browning in iWAT from lean control and fKO mice following 10-day treatment with CL316,243 (n = 6 mice per group). Target gene expression was normalized to TBP and presented as fold change over the mean relative expression from the PBS-injected group for each respective genotype. (**i**) Body weight measurements for control and fKO mice consuming high-fat diet while housed at thermoneutrality for 10 wks (n = 7–9 mice per group). (**j**) Tissue weight measurements for control and fKO mice after consuming high-fat diet while housed at thermoneutrality for 10 wks (n = 7–9 mice per group). Data presented as mean ± SEM with sample sizes listed above. The difference in means was analyzed using Student’s t-test where *P < 0.05 and **P < 0.01, ***P < 0.001, and ****P < 0.0001.

In order to understand why fKO promotes adiposity upon HFD feeding, indirect calorimetry experiments were performed. After acclimation to the cage system, the fKO mice had a significant reduction in their energy expenditure (Fig. 4a), suggesting that a lower metabolic rate is contributing to the larger fat mass observed in the fKO mice during obesity. Additionally, the fKO mice had a significant downward shift in their respiratory exchange ratio (Fig. 4b), suggesting a preference to use more lipids as an energy source. This could be related with the observation that despite high insulin and hepatic insulin resistance, the fKO mice did not develop steatosis, and had a trend towards increased plasma ketones when fasted (Supplemental Fig. S2). Interestingly, the increased body weight and lipid consumption are not due to increased food intake nor behavioral changes in the fKO mice (Fig. 4c,d). No changes in distance traveled or x-axis and y-axis beam breaks were observed in these mice (Fig. 4d), and the mice had a trend towards less daily food consumption (Fig. 4c).

Brown adipose tissue (BAT) is a significant contributor to global energy expenditure and upon stimulation accounts for an estimated 16% of the resting metabolic rate in humans^40^. Changes in the metabolic rate of this tissue would have large impacts on the basal metabolic rate of the animal as a whole. Despite the weight of BAT not being altered by fKO in diet-induced obese mice, H&E staining revealed gross morphological changes to the BAT in the fKO mice, which exhibited significant whitening of the tissue following HFD diet (Fig. 4e). Analysis of functional marker expression with qPCR showed that there was no change in uncoupling protein 1 (UCP1) expression, but Fgf21, Cox8b, and Elovl3 were upregulated in the BAT from the fKO mice (Supplemental Fig. S3). The upregulation of Fgf21 in the fKO BAT did not translate to altered plasma FGF-21 in mice with diet-induced obesity (Supplemental Fig. S4), which is likely attributable to the relatively higher secretion from the liver^41^.

The fKO BAT exhibited no significant reduction in the expression of genes involved in oxidation (Fig. 4f). However, there was a fourfold increase in the expression of Acc1, the enzyme that converts acetyl-CoA to malonyl-CoA, and a 2.5-fold increase in Fasn expression. Malonyl-CoA serves as a substrate for de novo lipogenesis and palmitate production. It has also been shown to inhibit beta oxidation through inhibition of carnitine palmitoyltransferase 1 (CPT1), the enzyme responsible for the influx of fatty acyl-CoA molecules into the mitochondria via the carnitine shuttle^42^. In addition to upregulation of Acc1, there is a trend towards the fKO BAT having reduced expression of Cpt1b, the CPT1 isoform expressed in BAT, skeletal muscle, and the heart. Together, these data suggest that the BAT of the fKO mice is whiter due to lipid flux shifting away from fatty acid oxidation and towards more de novo lipogenesis and lipid accumulation.

In addition to BAT, brown-like beige adipocytes are also significant contributors to basal metabolic rate. We observed that there is a significant reduction in Ucp1 and Pgc1α in the iWAT of lean, chow-fed fKO mice (Fig. 4g). To further determine whether deficits in browning and adaptive thermogenesis could contribute to the reduced energy expenditure in the fKO mice, beige adipocyte formation in the iWAT was analyzed following treatment with the β3-adrenregic receptor agonist, CL 316,243. β3-adrenregic receptor stimulation with CL316,243 promotes the transdifferentiation of white adipocytes to beige adipocytes, as opposed to de novo formation of beige adipocytes that differentiate from precursor cells following cold stimulation^43^. The iWAT from chow-fed fKO mice exhibited a blunted beiging capacity following 10 days of CL treatment at ambient temperature. The fKO mice had lower expression of the thermogenic markers Ucp1, Pgc1α, Cox8b, Elovl3, Cidea and the beige adipocyte marker Prdm16 compared to control mice injected with CL (Fig. 4h).

Surprisingly, the fKO iWAT did not have reduced thermogenic marker expression following 7 days of cold exposure. Instead, there was a significant increase in the expression of some of the thermogenic markers, including Ucp1 (Supplemental Fig. S4). This suggests that the ablation of CREB3L3 specifically limits the ability of subcutaneous adipocytes to undergo transdifferentiation into beige adipocytes following pharmacological stimulation, but does not reduce the ability for precursor cells to differentiate into beige cells upon cold exposure. In accordance with this, we found that the iWAT and eWAT from fKO mice exhibited strong trends towards increased Ucp1 expression following high-fat feeding (Supplemental Fig. S4). However, these differences were not significant and Western blotting revealed no differences in the abundance of UCP1 in the obese iWAT or eWAT (Supplemental Fig. S4). There was also no increase in the expression of markers of fatty acid oxidation in the eWAT, although there was a fivefold increase in PPARα expression in the obese iWAT (Supplemental Fig. S4). Additionally, we observed that lipolytic markers like Adrb3, lipoprotein lipase, and adipose triglyceride lipase (ATGL) were significantly downregulated and perilipin 2 was upregulated in the fKO eWAT following high-fat feeding. However, expression of these markers were unchanged in the iWAT (Supplemental Fig. S5) and obese fKO mice had no change in plasma free fatty acids after adipose lipolysis was stimulated with CL316,243. Since CREB3L3 promotes lipolysis in the liver^19^, this suggests a different role for CREB3L3 in adipose lipid metabolism.

To determine the tissue contributions to the larger body weight of the fKO mice following high-fat feeding, a cohort of mice were housed at thermoneutrality and fed a HFD for 14 weeks. Ambient temperature is sufficient to increase the activity of brown fat, but brown fat activity is reduced when mice are housed at thermoneutrality and thermogenesis is not needed to maintain body temperature^44^. The differences in body weight (Fig. 4i) and adipose tissue size (Fig. 4j) between the control and fKO mice disappeared when the mice were housed at thermoneutrality. This suggests that the enhanced body weight and enlarged adipose tissues in the fKO mice during obesity (Fig. 2a–c) are temperature-dependent and are caused by diminished energy expenditure in the fKO brown fat.

### Increased expression of CREB3L3 in subcutaneous adipose tissue prevents diet-induced obesity

**Figure 5:**
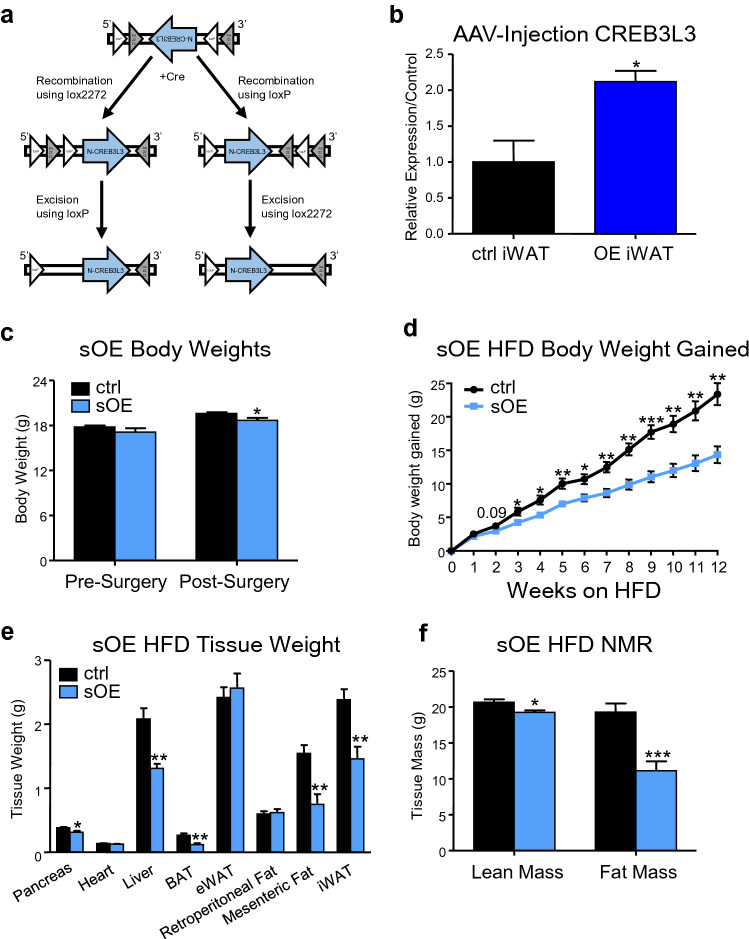
Increased expression of CREB3L3 in subcutaneous adipose tissue prevents diet-induced obesity. (**a**) Schematic depicting the recombination and inversion of CREB3L3 within the flip-excision (FLEx) AAV in the presence of Cre recombinase. (**b**) Quantitative PCR for the expression of CREB3L3 message in inguinal fat pads harvested two weeks following injection with the CREB3L3 FLEx AAV in Adiponectin-Cre (sOE) or littermates lacking the Cre BAC (ctrl) (n = 3–4 mice per group). CREB3L3 expression was normalized to TBP and presented as fold change over ctrl. (**c**) Body weight measurements of sham ctrl and sOE mice 2 wks after injection with the CREB3L3 FLEx AAV (n = 6–9 mice per group). (**d**) Measurements of the body weight gained by sham control and subcutaneous overexpression (sOE) mice after high-fat feeding (n = 6–9 mice per group). (**e**) Tissue weights measurements for sham control and sOE mice after high-fat feeding (n = 6–9 mice per group). (**f**) Body composition of sham and sOE mice following high-fat feeding measured by NMR (n = 6–9 mice per group). Data presented as mean ± SEM with sample sizes listed above. The difference in means was analyzed using Student’s t-test where *P < 0.05, **P < 0.01, and ***P < 0.001.

Since CREB3L3 message is selectively downregulated in the iWAT of obese mice (Fig. 1a,b,e), this natural knockdown following high-fat feeding could limit our ability to make inferences about the role of this transcription factor in the subcutaneous adipose biology of the fKO mice. To better study the role of this transcription factor in obese iWAT, we aimed to reintroduce CREB3L3 into this tissue by iWAT-specific overexpression and subsequent examination of the metabolic consequences. The processed form of CREB3L3 (smaller band in OE iWAT, Fig. 1f) was overexpressed in the iWAT of Adipo-Cre mice via direct injection of a CREB3L3-AAV (adeno-associated virus), where the CREB3L3 reading frame is inverted and housed between loxP and lox2272 sites (Fig. 5a). Using this flip-excision (FLEx) technology^45^, Cre recombination results in the inversion of the reading frame and forced overexpression only in the adipose tissue which was directly injected with the AAV (Fig. 5b).

Despite having no significant difference in their body weights before the surgery, the mice with subcutaneous overexpression (sOE) weighed less than sham littermate controls lacking the Adipo-Cre BAC 10 days post-surgery (Fig. 5c). This difference was exacerbated during high-fat feeding. Strikingly, increased expression of CREB3L3 reduced body weight gain in mice with diet-induced obesity. The sOE mice were 14.4 g heavier following 12 weeks on HFD. Control mice that were also injected with the virus gained 23.4 g over the same time period (Fig. 5d). This difference in body weight was due to significant reductions in iWAT, BAT, mesenteric fat, pancreas, and liver size in the sOE mice (Fig. 5e). Interestingly, there were no changes in the eWAT and retroperitoneal fat, although this could be due to compensatory fat storage (Fig. 5e). The reduction in iWAT size does not appear to be due to reduced adipocyte formation in this tissue, as C/EBPβ was the only adipogenic marker that exhibited a slight downregulation in the sOE iWAT. Overall, the sOE mice had a dramatic reduction in their fat mass, in addition to a small reduction in lean mass (Fig. 5f).

### Subcutaneous adipose overexpression of CREB3L3 prevents obesity-associated metabolic dysfunction

**Figure 6:**
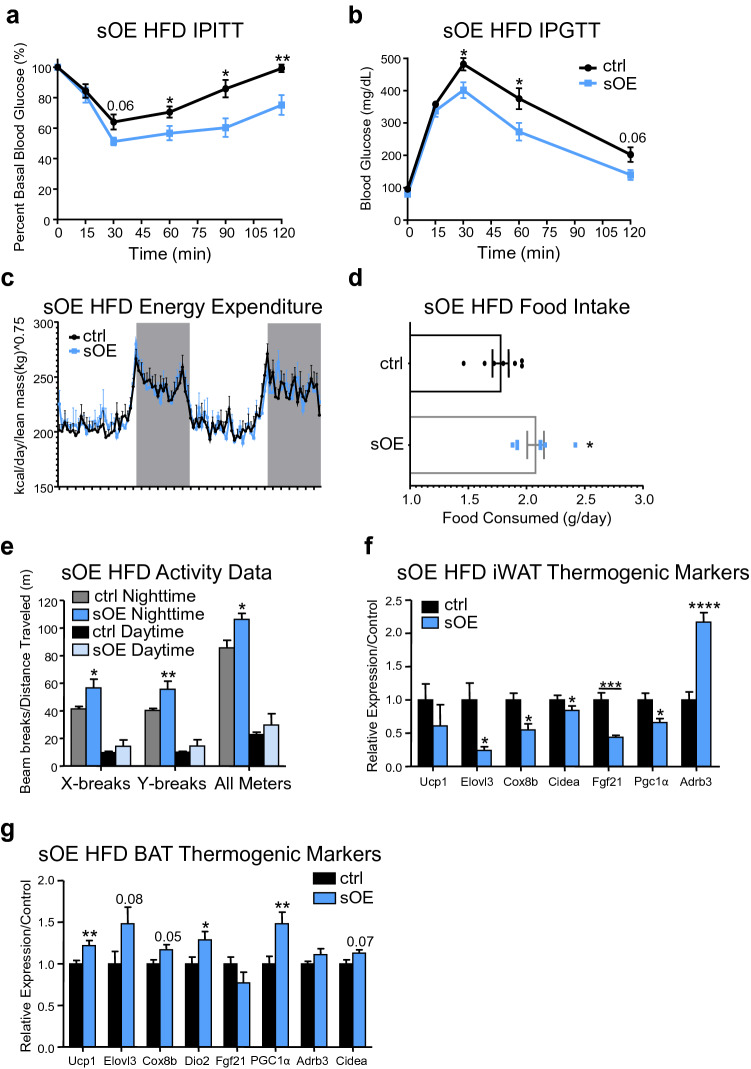
Subcutaneous adipose overexpression of CREB3L3 prevents obesity associated metabolic dysfunction. (**a**, **b**) Intraperitoneal (**a**) insulin and (**b**) glucose tolerance tests performed with sham control and sOE mice (n = 5–7 mice per group). (**c**) Energy expenditure of sham ctrl and sOE mice fed high-fat diet (n = 6–9 mice per group). Energy expenditure was normalized to the animal’s lean mass. The curve was analyzed using two-way ANOVA. The p value for the column factor (genotype) was 0.4992. (**d**) Average daily consumption of high-fat diet while ctrl and sOE mice were in metabolic cages in metabolic cages (n = 6–8 mice per group). (**e**) Activity measurements of sham ctrl and sOE mice fed a high-fat diet. Data presented as x- and y-axis beam breaks and total movement per day following acclimation period (n = 5–8 mice per group). (**f**, **g**) Quantitative PCR for markers of thermogenesis in **(f)** iWAT or **(g)** brown adipose tissue from ctrl and sOE mice following high-fat diet (n = 6–8 mice per group). Target gene expression was normalized to TBP and presented as fold change over control. Data presented as mean ± SEM with sample sizes listed above. The difference in means was analyzed using Student’s t-test where *P < 0.05, **P < 0.01, ***P < 0.001, and ****P < 0.0001.

Consistent with their leaner phenotype, the sOE mice had improved insulin sensitivity (Fig. 6a), in addition to having improved glucose tolerance compared to the sham control mice (Fig. 6b). The improved body weight and insulin sensitivity of the sOE mice are not due to increased energy expenditure (Fig. 6c) or reduced food intake (Fig. 6d). The improved insulin sensitivity also occurred despite increased iWAT expression of the pro-inflammatory cytokines MCP1, TNFα, and interleukin 1 beta (IL-1β) (Supplemental Fig. S6).

Changes in body composition (Fig. 5d–f) and insulin sensitivity may stem from the increased physical activity observed in the sOE mice (Fig. 6e). Changes in iWAT energy expenditure do not appear to contribute to the reduced body weight of the sOE mice. Expression of thermogenic markers like PGC1α, Cidea, Cox8b, Elovl3, and Fgf21 were significantly reduced in the sOE iWAT, and there was a trend towards Ucp1 expression being reduced as well (Fig. 6f). Interestingly, thermogenic marker expression was increased in the brown fat from the sOE mice, with PGC1α and Ucp1 being significantly upregulated (Fig. 6g). This increase in thermogenic gene expression in the brown fat could also contribute to the ability of the sOE mice to resist diet-induced obesity.

## Discussion

Body weight regulation has become a crucial public health issue over the past few decades and as of 2015–2016, 39.6 percent of American adults were obese^46^. Due to the substantial contributions of brown and beige fat to body weight reduction as both high energy-usage tissues and as endocrine organs, they have been the targets of recent therapeutic investigation^12^. Additionally, the chronic low-level inflammation that occurs in the adipose tissue during obesity spurs the development of metabolic syndrome and a host of deadly comorbidities^47^. While increased adiposity typically leads to deleterious changes in whole-body metabolism, the expansion of different adipose tissues does not contribute equally to the development of metabolic dysfunction. It is well-known that expansion of visceral adipose tissue is associated with metabolic dysfunction, whereas subcutaneous adipose expansion might even be protective^48^. It has been shown that visceral and subcutaneous adipocytes are derived from different developmental origins^49^, but studies investigating the differences between mature subcutaneous and visceral adipocytes are lacking.

In this study, we discovered that adipose expression of CREB3L3 is modulated by obesity. Previous studies reported the expression of CREB3L3 in a variety of tissues, but did not examine the expression of this ER-bound transcription factor in adipose tissues^17,26,27^. Here we report that CREB3L3 is not a liver-specific protein, and both CREB3L3 message and protein can be detected in the subcutaneous, brown, and multiple visceral adipose tissues (Fig. 1e,f). In addition to being present in the adipose tissue, we also found that CREB3L3 is selectively downregulated in the subcutaneous fat during obesity, while the expression in the visceral fat is largely unchanged (Fig. 1a–d). This differential expression pattern was corroborated by the correlations with body fat mass (Table 1). Future investigations will study the mechanism responsible for this differential expression. The selective downregulation of CREB3L3 in obese subcutaneous fat and the negative association between subcutaneous CREB3L3 and body fat mass led us to hypothesize that this downregulation of CREB3L3 in obese subcutaneous fat could contribute to the divergent metabolic profiles of subcutaneous and visceral fat during obesity.

Previous studies have shown that the whole-body knockout of CREB3L3 leads to reduced body weight and adipose tissue size in mice fed an atherogenic high-fat diet. However, this is due to reduced lipoprotein lipase activity and defects in lipid uptake into the tissues^18^. Here, we unmasked the contributions of CREB3L3 to both subcutaneous and visceral adipose biology using in vivo fat-specific ablation of this transcription factor in mice of a mixed genetic background. While their mixed background constitutes a limitation of our fat-specific ablation studies, it does model the heterogeneity of patient populations better than single-strain mouse models. In our fat-specific knockout mice, we found that adipose ablation of CREB3L3 promotes diet-induced obesity (Fig. 2a–c). The subcutaneous fat from these mice has a reduced ability to undergo browning in response to a β3-adrenergic agonist (Fig. 4h) and the brown fat exhibited reduced energy expenditure and lipid consumption upon high-fat feeding (Fig. 4e,f). Given the preserved browning potential of the iWAT in response to cold (Supplemental Fig. 4) and the temperature-dependence of the obesity-promoting phenotype of the fKO mouse (Fig. 4i,j), it appears that altered brown fat metabolism is the main contributor to the obesigenic effect of adipose CREB3L3 ablation.

In the visceral fat, the ablation of CREB3L3 lead to enhanced macrophage infiltration, altered adipokine expression, and increased pro-inflammatory cytokines in the obese state (Fig. 3f,g,k). While the subcutaneous fat also experienced enhanced macrophage infiltration (Supplemental Fig. S3), there were no changes in adipokine or pro-inflammatory cytokine expression (Fig. 3h). This occurred despite the shift towards the fKO tissue having more hypertrophic adipocytes (Fig. 3j). Additionally, overexpression of CREB3L3 in the iWAT upregulated pro-inflammatory cytokines (Supplemental Fig. S6). This suggests that CREB3L3 could differently contribute to the inflammatory response in visceral and subcutaneous fat, which requires further investigation.

One of the reasons we do not fully understand the differences between subcutaneous and visceral adipocytes is the difficulty in producing fat-depot specific genetic manipulations. This is due to the lack of depot-specific genes from which the promoter can be used to drive the expression of Cre recombinase. In this study, we describe the creation of a subcutaneous fat-specific overexpression model via injection of a CREB3L3 FLEx-AAV directly into the subcutaneous fat pads of mice expressing Adiponectin-Cre (Fig. 5a). Adipose-specific viral transductions have proven to be difficult due to the tendency for viral vectors to transduce non-adipocyte cell types and the size of the adiponectin promoter limits its cloning potential in AAVs. Previously, groups have cloned “mini” aP2 promoters^50^ or micro RNAs into the AAV vectors to prevent off-target expression^51^. Our system inverts the reading frame of interest to prevent expression in non-adipocytes (Fig. 5a). Since the element needed for adipocyte specificity is programmed into the adipocytes themselves, the investigator is afforded more cloning flexibility and does not need to accommodate any adipose-specific elements into the AAV. To our knowledge, this is the first demonstration of using a viral vector in conjunction with adiponectin-Cre to manipulate gene expression in a specific fat depot.

Using this overexpression system, we created a CREB3L3 subcutaneous fat-specific overexpression model (sOE). These mice were resistant to diet-induced obesity, further demonstrating the importance of CREB3L3 in body weight regulation. One of the unique features of our overexpression system is the degree to which our gene of interest is overexpressed. Many transgenic and viral-induced overexpression systems force expression to levels more than an order of magnitude higher than endogenous levels. This supraphysiological expression can overload cells and shift cellular energetics and signaling^52^, our FLEx-AAV system induced a more modest twofold increase in CREB3L3 expression (Fig. 5b). The robustness of the observed sOE phenotype with such a relatively small change in gene expression underpins the ability of CREB3L3 to modulate body weight. It also suggests that the fourfold lower subcutaneous fat expression of CREB3L3 (Fig. 1c) could contribute to the body weight dysregulation of obese patients.

Interestingly, the sOE mice do not exhibit increased energy expenditure (Fig. 6c), despite being significantly more active than Cre-null CREB3L3 FLEx-AAV-injected control mice (Fig. 6e). This may be due to one of the limitations of our approach to inducing overexpression via AAV injection. The AAV virus was injected into mice at 5 weeks of age when the inguinal fat depot is still developing and expanding. As the inguinal fat continues to expand in these adolescent mice, especially as they undergo high-fat feeding, many more adipocytes differentiate from adipose-derived stem cells. Adipocytes do not proliferate, but do turnover due to adipocyte death, especially when under dietary stress^53^. Therefore, the proportion of adipocytes that overexpress our gene of interest likely decreases over the course of dietary treatment. After monitoring the body weight during 12 weeks of high-fat feeding, then performing tolerance tests, the pool of adipocytes in the sOE inguinal fat that still overexpressed CREB3L3 when the sOE mice underwent indirect calorimetry measurements was likely diminished, which is demonstrated by the regressed CREB3L3 expression when tissues were subsequently harvested from these mice (Supplemental Fig. S6). Despite these limitations, the sOE mice maintained their resistance to diet-induced obesity, and still exhibited reduced fat mass and improved glucose tolerance and insulin sensitivity after the 12 weeks of high-fat feeding, despite consuming more food than controls (Fig. 6d). This may be due to increased brown fat thermogenesis (Fig. 6g). We postulate that the correlation between inguinal CREB3L3 expression and energy expenditure in brown fat in our mouse models suggests that CREB3L3 could potentially drive crosstalk between the tissues. Alternatively, the increased brown fat thermogenesis in the sOE mice could be compensatory for the reduced browning of the iWAT (Fig. 6f). Regardless, further investigation into this association is required.

The resistance of sOE mice to diet-induced obesity, when coupled with enhanced diet-induced obesity in the fKO mice, demonstrated the important role that CREB3L3 plays in body weight and fat mass regulation. Additionally, the enhanced pro-inflammation cytokine expression within the epididymal fat of the fKO mice during obesity suggests that CREB3L3 could play a role in limiting the extent of inflammation within obese visceral fat. Further investigation into the role of CREB3L3 in the regulation of adipose inflammation is also needed. Together, through its contributions to regulating both adiposity and visceral inflammation, we have identified the importance of CREB3L3 in the transcriptional control of adipose behavior during obesity. Given its ability to modulate body weight during obesity, adipose CREB3L3 could offer a potential weight loss target to help curb the obesity epidemic.

## Methods

### Contact for reagent and resource sharing

Further information and requests for resources and reagents should be directed to and will be fulfilled by the Lead Contact, Chong Wee Liew (cwliew@uic.edu).

### Experimental model and subject details

#### Animals

Mice were housed at environmentally controlled conditions with a 12 h light/dark cycle with free access to standard rodent pellet food and water, with changes in diet or environmental temperature specified. Animal protocols were approved by the Animal Care and Use Committee (IACUC) of the University of Illinois at Chicago. Animal care was provided in accordance with institutional guidelines. C57BL/6J, *ob/ob*, and Adiponectin-Cre mice were obtained from the Jackson Laboratory (Bar Harbor, ME, USA). Adiponectin-Cre mice used in this study were on a C57BL/6J background, and the CREB3L3 floxed mice were on a mixed (129 Sv/CD-1) background. All animal studies were performed in compliance with the ARRIVE guidelines.

#### Subjects for CREB3L3 adipose tissue expression

In a cross-sectional study of 636 individuals (433 women, 203 men; BMI range: 14.7–88.8 kg/m^2^, age range: 18–90 years), we investigated *CREB3L3* mRNA expression in paired abdominal omental and subcutaneous (SC) Adipose tissue samples collected during elective laparoscopic abdominal surgery as described^54^. Adipose tissue was immediately frozen in liquid nitrogen and stored at − 80 °C. The study was approved by the Ethics Committee of the University of Leipzig (approval no: 159-12-21052012) and performed in accordance with the declaration of Helsinki. All subjects gave written informed consent before taking part in this study. Measurement of body composition and metabolic parameters was performed as described previously^54,55^. The magnitude and significance of their correlation to the expression of CREB3L3 in omental and abdominal subcutaneous fat was measured using Pearson’s correlation coefficient.

#### Generation of *CREB3L3* fat-specific knockout mouse model

CREB3L3 floxed mice were rederived from frozen embryonic stem cells received from Stephen Duncan^27^. CREB3L3^flox/flox^ mice on a mixed (129 Sv/CD-1) background were crossed with Adiponectin-Cre (Adipo-Cre) mice on a C57BL/6j background to generate a fat-specific knockout (fKO) of CREB3L3. CREB3L3^flox/flox^ mice containing the Adipo-Cre allele were bred with CREB3L3^flox/flox^ littermates lacking the Adipo-Cre allele to generate the mice for the fKO experiments. CREB3L3^flox/flox^ mice with the Adipo-Cre allele were termed fKO mice while littermates lacking the Cre allele were used as control mice in fKO experiments.

#### Body weight study

During diet-induced obesity studies, mice were fed chow diet (17% fat, 25% protein and 58% carbohydrate by kcal; #7012, Envigo, Indianapolis, Indiana, USA) until 6 weeks of age. At this time, the mice were placed on high-fat diet (60% fat, 20% protein, and 20% carbohydrate by kcal; D12492, Research Diets, New Brunswick, NJ, USA) for the remainder of the experiment. Body weight was measured weekly until 16 weeks of age. For the measurement of body weight at thermoneutrality, the mice were housed in an environmental chamber (Powers Scientific, Pipersville, PA, USA) at 30 degrees Celsius at the start of high-fat feeding.

#### Subcutaneous fat expression of CREB3L3 in vivo

To overexpress CREB3L3 in the subcutaneous adipose tissue, a flip-excision (FLEx) adeno-associated virus (AAV) encoding the inverted reading frame of the processed form of CREB3L3 was created. The inverted reading frame was flanked by both loxP and lox 2272 sites, which will cause the reading frame to be inverted upon interaction with Cre recombinase^45^. This scheme is designed to prevent off-target overexpression. The AAV (serotype 8, 4 × 10^11^ vg/mL, Viral Core, Boston Children’s Hospital, Boston, MA, USA) was injected directly into both inguinal fat pads of Adiponectin-Cre mice and littermate controls lacking the Adiponectin BAC at 5wks of age. Each fat pad received 8 injections of 3uL dispersed evenly across the length of the tissue. The mice recovered from surgery for 10 days before being placed on high-fat diet for the remainder of the experiment. Body weight was measured weekly until 20 weeks of age.

#### *CREB3L3* mRNA expression in human visceral and subcutaneous adipose tissue

RNA from human adipose tissue was prepared as described previously^54^. The RNA was extracted using RNeasy Lipid tissue Mini Kit (Qiagen, Hilden, Germany). Quantity and integrity of RNA was monitored with NanoVue plus Spectrophotometer (GE Healthcare, Freiburg, Germany). 1 µg total RNA from SC and Vis adipose tissue were reverse transcribed with standard reagents (Life technologies, Darmstadt, Germany). cDNA was then processed for TaqMan probe-based quantitative real-time polymerase chain reaction (qPCR) using the QuantStudio 6 Flex Real-Time PCR System (Life technologies, Darmstadt, Germany). Expression of *CREB3L3* was calculated by standard curve method and normalized to the expression of *hypoxanthine guanine phosphoribosyltransferase 1* (*HPRT1*) as a housekeeping gene. The probes (Life technologies, Darmstadt, Germany) for *CREB3L3* (Hs00261701_m1) and *HPRT1* (Hs01003267_m1) span exon-exon boundaries to improve the specificity of the qPCR.

#### Measurement of mouse lipid contents

Lipids were extracted from liver samples using Folch solution composed of a 2:1 (vol/vol) mixture of chloroform/methanol as described previously^56^. Lipids were solubilized in 10% Triton X-100 before evaporation. Non-esterified fatty acid (Fujifilm/Wako, Lexington, MA, USA), cholesterol (Stanbio, Boerne, TX, USA), and triglyceride content (Stanbio, Boerne, TX, USA) were measured in hepatic extracts and plasma samples using colorimetric assays.

#### Food intake, energy expenditure, physical activity, and body composition

Food intake, oxygen consumption, carbon dioxide production, energy expenditure, and physical activity were measured using the Promethion System (Sable Systems International, Las Vegas, NV, USA). The energy expenditure was normalized to lean body mass. The respiratory exchange ratio (RER) was calculated using the VCO2/VO2 ratio from the gas exchange data. Body composition (lean and fat mass) was estimated using NMR (Bruker minispec LF50, Billerica, MA, USA).

#### Physiological studies and histological analyses

Blood glucose was measured using an automated glucose monitor (Glucometer Elite, Bayer, Bayer AG, Leverkusen, Germany). Tolerance tests were performed as described previously^57^. Briefly, mice underwent a 16 h overnight or 2 h morning fast, before the glucose (IPGTT) and insulin (IPITT) tolerance tests, respectively. During experiments with KO mice, mice were injected intraperitoneally (IP) with a bolus of 2 g/kg dextrose (Hospira, Lake Forest, IL, USA) for IPGTT or 1.5U/kg insulin (Eli Lilly, Indianapolis, IN, USA) for IPITT. During forced overexpression studies, 1U/kg insulin was used for IPITT. For fasting and refeeding study, mice underwent a 16 h overnight fast before being sacrificed or refed for 4 h or 8 h prior to being sacrificed. For the in vivo lipolysis assay, mice underwent a 6 h morning fast before injection with 1 mg/kg of the β3-adrenergic receptor agonist CL 316,243 (Tocris Bioscience, Minneapolis, MN, USA) and subsequent blood collection from the tail one hour after injection. For histological analyses, mice were sacrificed, and tissues were excised, weighed, and processed for immunohistochemistry as described previously^58^. White adipose tissues were fixed for 48 h in formalin and immediately processed. Tissues were sectioned by the Human Tissue Resource Center (University of Chicago, Chicago, IL, USA).

#### Insulin stimulation assay

Mice were fasted overnight for 16 h before IP injection of PBS (baseline) or 1U insulin. Tissues were quickly harvested five minutes after injection. Western blotting was performed to quantify the level of Akt phosphorylation in the liver and eWAT.

#### RNA extraction and real time PCR

Total RNA was isolated from tissues and mature adipocytes using Trizol reagent (Invitrogen, Carlsbad, CA, USA) and the Direct-zol Kit (Zymo, Irvine, CA, USA). Mature adipocytes were isolated from inguinal and epididymal adipose tissue following digestion with collagenase Type 1 (2 mg/mL Worthington Biochemical, Lakewood, NJ, USA) as described previously^15^. cDNA was generated from 2 µg of total RNA using the High Capacity cDNA Reverse Transcription Kit (Invitrogen, Carlsbad, CA, USA) using random hexamer primers, following the manufacturer’s instructions. The resulting cDNA was diluted to a concentration of 10 ng/µL and 1.67 µL was aliquoted for each 6.67 µL qPCR reaction (SYBR Green, Bio-Rad, Hercules, CA, USA). Reactions contained primers (Integrated DNA Technologies, Coralville, IA, USA) at a concentration of 300 nM each, and 600 nM for Ucp1 in white adipose tissue. PCR reactions were run in duplicate and quantitated using the ViiA^TM^7 Real-Time PCR system (Applied Biosystems, Foster City, CA, USA). Results were normalized to *Beta-actin (B-actin), Ribosomal protein L19 (L-19),* or *TATA box binding protein* (*TBP*) expression and expressed as arbitrary units or fold change. Primer sequences are listed in Supplementary Table S1.

#### Western blotting

Tissues were homogenized in radioimmunoprecipitate (RIPA) buffer with protease inhibitor and phosphatase inhibitor cocktail 2 and 3. Lysates were denatured by boiling in Laemli buffer with 2-mercaptoethanol and 20–80 μg were loaded onto 8–10% SDS-PAGE gels. Proteins were transferred onto nitrocellulose membranes and developed using multiple exposures to determine signal linearity. Antibodies used for immunoblotting were CREB3L3 (Kezhong Zhang, Wayne State University), S473-Akt (CST, 7074S, Danvers, MA, USA), pan Akt (CST, 7074S, Danvers, MA, USA), UCP1 (Abcam, ab10983, Cambridge, UK) and β-actin (Proteintech, Rosemont, IL, USA).

#### ELISAs & multiplex

Enzyme-linked immunosorbent assays (ELISA) were performed to measure the fed and fasting plasma concentrations of insulin (Crystal Chem USA, Elk Grove, IL, USA), adiponectin (RayBiotech Life, Peachtree Corners, GA, USA), and FGF-21 (BioVendor, Asheville, NC, USA) following high-fat feeding. High-fat fed plasma concentrations of leptin and MIP-2 were measured via magnetic luminex assay (R&D Systems Inc., Minneapolis, MN, USA).

#### CL316,243 treatment

Mice were IP injected daily with 1 mg/kg of the β3-adrenergic receptor agonist CL 316,243 (Tocris Bioscience, Minneapolis, MN, USA) or PBS (Corning, Corning, NY, USA) daily for 10 days, starting at 11 weeks of age.

### Quantification and statistical analysis

#### Mouse model analyses

All data are presented as the mean ± standard error of mean (SEM) and were analyzed by unpaired two-tailed Student’s t-test, with an α of 0.05. Gas exchange data from the metabolic cages were analyzed by two-way ANOVA. Sample sizes were determined using prior characterization experiments for the mouse models used for this study. Mice were randomized to treatment in a blinded manner when possible.

#### Adipocyte size quantification

Tissue sections from eWAT and iWAT were stained with anti-caveolin-1 antibody (Abcam, Cambridge, United Kingdom) and images of the adipocyte outlines were taken. Adipocyte areas were measured using a pipeline developed in CellProfiler ^59^. Cell area frequencies were determined and graphed using GraphPad Prism 6 (Graphpad Software, San Diego, CA, USA).

#### Crown-like structure quantification

Representative images were taken from H&E-stained eWAT and iWAT tissue sections using the Zeiss Observer Z1 (Zeiss, Oberkochen, Germany) microscope. The observer was blinded and counted the number of crown-like structures within each field of view. Images were grouped by genotype and analyzed using student’s T-test^60^.

## Supplementary Information

Supplementary Information.
