# Author Correction: Adipose expression of CREB3L3 modulates body weight during obesity

**DOI:** 10.1038/s41598-021-99603-3

**Published:** 2021-10-05

**Authors:** Maximilian A. McCann, Yanliang Li, Marcos Muñoz, Victoria Gil, Guifen Qiang, Jose Cordoba-Chacon, Matthias Blüher, Stephen Duncan, Chong Wee Liew

**Affiliations:** 1 Department of Physiology & Biophysics, University of Illinois at Chicago, Chicago, IL USA; 2 Department of Endocrinology, Huashan Hospital, Fudan University, Shanghai, China; 3 Department of Medicine, Section of Endocrinology, Diabetes and Metabolism, University of Illinois at Chicago, Chicago, IL USA; 4 Helmholtz Institute for Metabolic, Obesity and Vascular Research (HI-MAG) of the Helmholtz Zentrum München at the University of Leipzig and University Hospital Leipzig, Leipzig, Germany; 5 Department of Regenerative Medicine & Cell Biology, Medical University of South Carolina, Charleston, SC USA; 6 Department of Physiology & Biophysics, College of Medicine, University of Illinois at Chicago, 835 S Wolcott Ave, M/C 901, MSB, E-202, Chicago, IL 60612 USA; 7 Present Address: State Key Laboratory of Bioactive Substances and Functions of Natural Medicines, Institute of Materia Medica, Chinese Academy of Medical Sciences and Peking Union Medical College, Beijing, China

Correction to: *Scientific Reports* 10.1038/s41598-021-98627-z, published online 29 September 2021

The Acknowledgements section in the original version of this Article was incomplete.

“The authors thank Kezhong Zhang of Wayne State University for providing the anti-CREB3L3 antibody. The work was supported by R00 DK090210, R01 DK109015, University of Chicago DRTC (DK020595) Pilot & Feasibility award, Central for Society for Clinical and Translational Research Early Career Development Award and UIC Startup fund (C.W.L.); M.A.M. was supported by an American Heart Association Predoctoral Fellowship (Grant No. 16PRE30190011). M.B. was funded by the Deutsche Forschungsgemeinschaft: Collaborative Research Center SFB1052, project B1.”

now reads:

“The authors thank Kezhong Zhang of Wayne State University for providing the anti-CREB3L3 antibody. The authors also acknowledge the Research Open Access Publishing (ROAAP) Fund of the University of Illinois at Chicago for financial support towards the open access publishing fee for this article. The work was supported by R00 DK090210, R01 DK109015, University of Chicago DRTC (DK020595) Pilot & Feasibility award, Central for Society for Clinical and Translational Research Early Career Development Award and UIC Startup fund (C.W.L.); M.A.M. was supported by an American Heart Association Predoctoral Fellowship. M.B. was funded by the Deutsche Forschungsgemeinschaft: Collaborative Research Center SFB1052, project B1.”

The original Article has been corrected.

