# Supplementary material for: Adipose expression of CREB3L3 modulates body weight during obesity
Source: Sci Rep. 2021 Sep 29;11:19400. doi: 10.1038/s41598-021-98627-z (PMC8481296; doi:10.1038/s41598-021-98627-z)
Supplement: Supplementary file 1 — Supplementary Information. [file 41598_2021_98627_MOESM1_ESM.pdf]

Adipose expression of CREB3L3 modulates body weight during obesity

Maximilian A. McCann[1], Yanliang Li[1,2], Marcos Muñoz[1], Victoria Gil[1], Guifen Qiang[1,3], Jose Cordoba-Chacon[4], Matthias Blüher[5], Stephen Duncan[6], Chong Wee Liew*[1]

[1]Department of Physiology & Biophysics, University of Illinois at Chicago, Chicago, IL, USA
[2]Department of Endocrinology, Huashan Hospital, Fudan University, Shanghai, China
[3]State Key Laboratory of Bioactive Substances and Functions of Natural Medicines, Institute of Materia Medica, Chinese Academy of Medical Sciences and Peking Union Medical College, Beijing, China (Current affiliation)
[4]Department of Medicine, Section of Endocrinology, Diabetes and Metabolism, University of Illinois at Chicago, Chicago, IL, USA
[5]Helmholtz Institute for Metabolic, Obesity and Vascular Research (HI-MAG) of the Helmholtz Zentrum München at the University of Leipzig and University Hospital Leipzig, Leipzig, Germany
[6]Department of Regenerative Medicine & Cell Biology, Medical University of South Carolina, Charleston, SC, USA

Corresponding author:
Chong Wee Liew PhD
Department of Physiology & Biophysics
College of Medicine
University of Illinois at Chicago
835 S Wolcott Ave, M/C 901, MSB, E-202, Chicago, IL 60612
Telephone: 1-312-413-1086; Fax: 1-312-996-1414
E-mail: cwliew@uic.edu

Figure S1

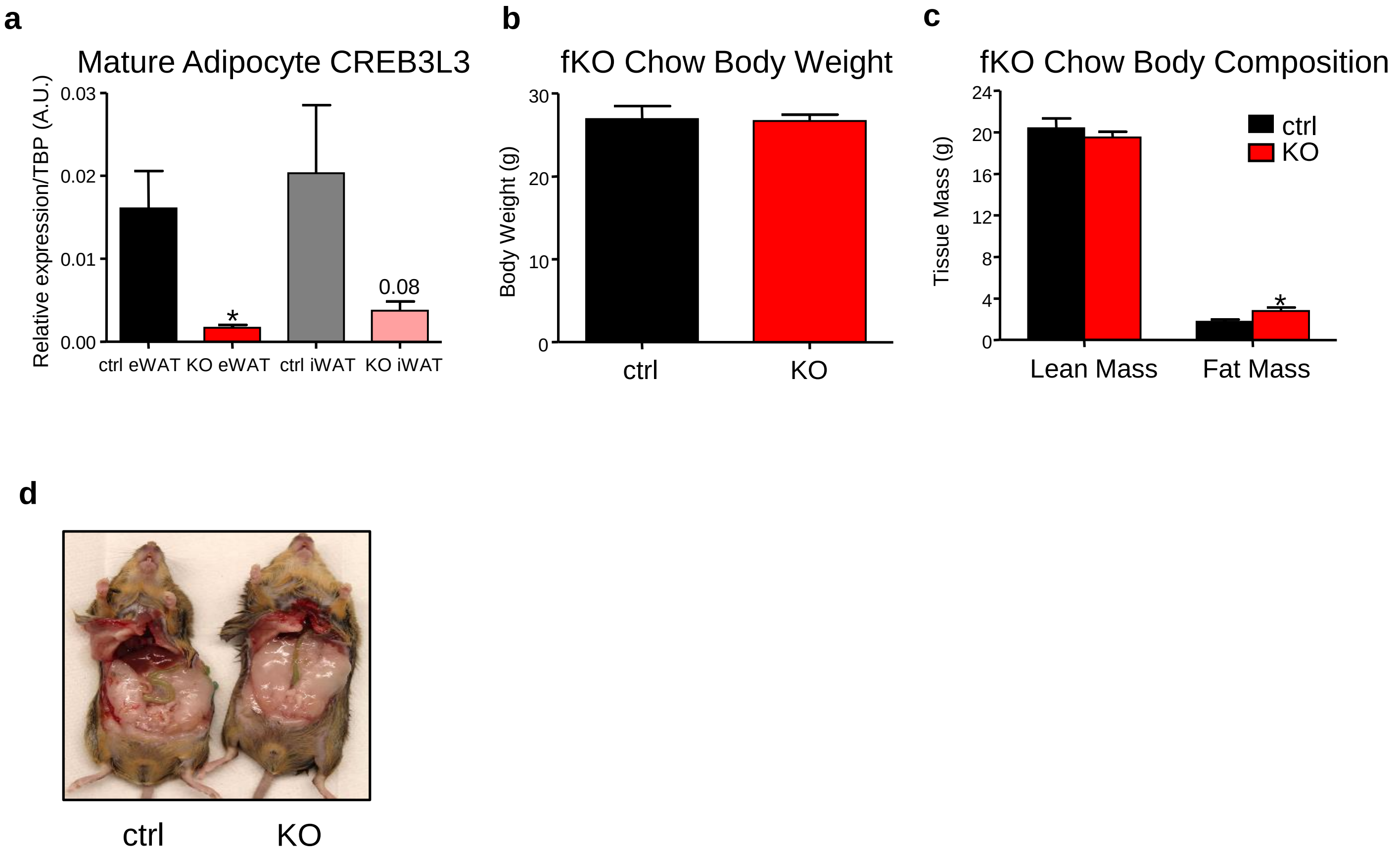

Figure S1. Adipose ablation of CREB3L3 enhances fat mass, but not body weight in mice fed a chow diet
(**a**) Quantitative PCR for the expression of CREB3L3 message in mature adipocytes isolated from digested epididymal (eWAT) and inguinal (iWAT) white adipose tissue (n= 4-5 mice per group). CREB3L3 expression was normalized to TBP housekeeping gene.
(**b-c**) Body weight and body composition NMR measurements of chow-fed control and fKO mice at 22 wks of age (n=7-9 mice per group).
(**d**) Representative image of control and fKO mice following high-fat feeding.
Data presented as mean +/- SEM. The difference in means was analyzed using Student’s t-test where *P<0.05.

Figure S2

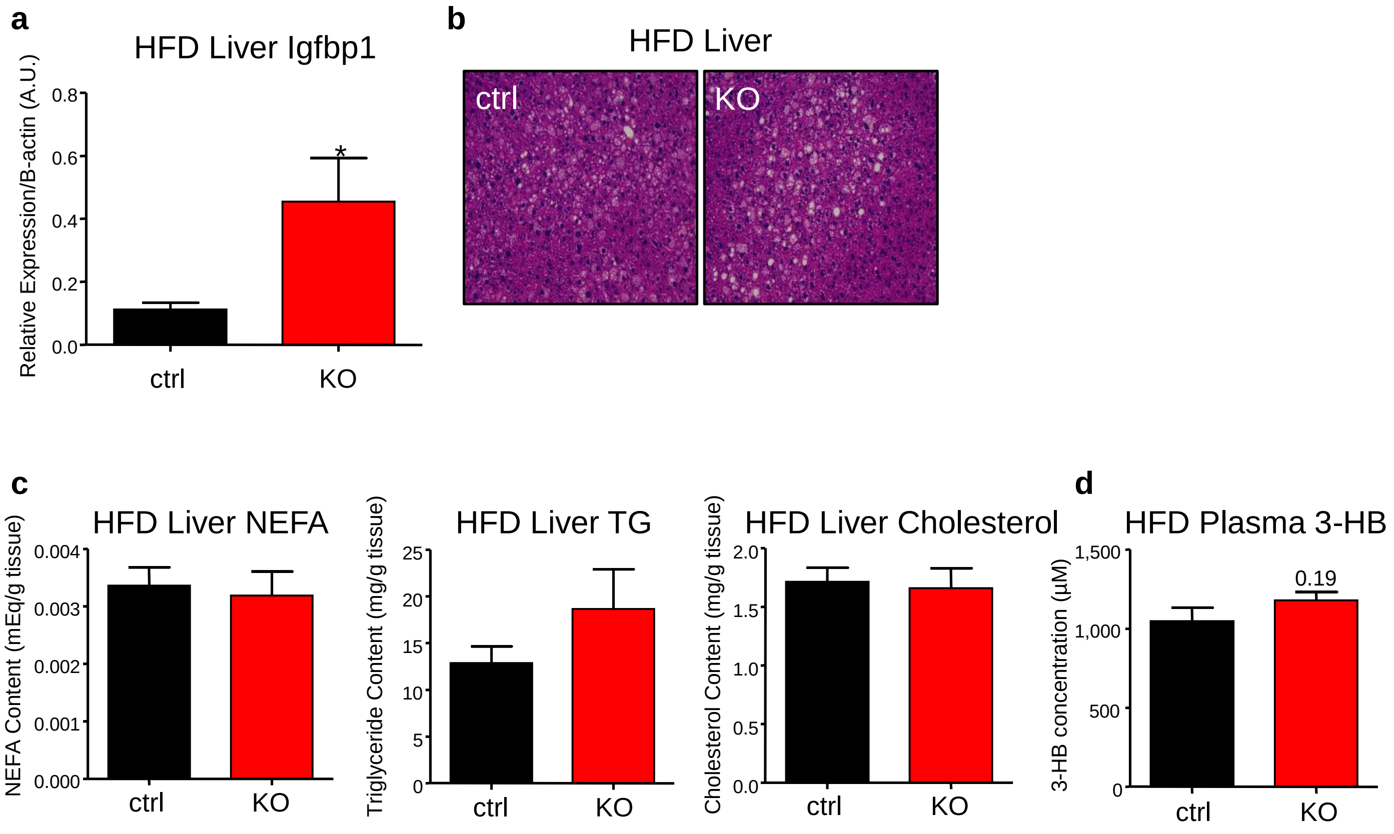

Figure S2. CREB3L3 fKO does not induce hepatic steatosis
(**a**) Quantitative PCR for insulin-like growth factor binding protein 1 (Igfbp1) in control and fKO livers following high-fat feeding (n=5 mice per group). Igfbp1 expression was normalized to B-actin housekeeping gene.
(**b**) Representative images of H&E-stained liver sections following high-fat feeding.
(**c**) Quantification of non-esterified fatty acids (NEFA), triglycerides (TG), and cholesterol from liver extracts following high-fat feeding using colorimetric assays (n=7-8 mice per group).
(**d**) Quantification of fasted plasma 3-hydroxybutyrate (3-HB) following high-fat feeding using colorimetric assay (n=7-8 mice per group). Data presented as mean +/- SEM. The difference in means was analyzed using Student's t-test where *P<0.05.

Figure S3

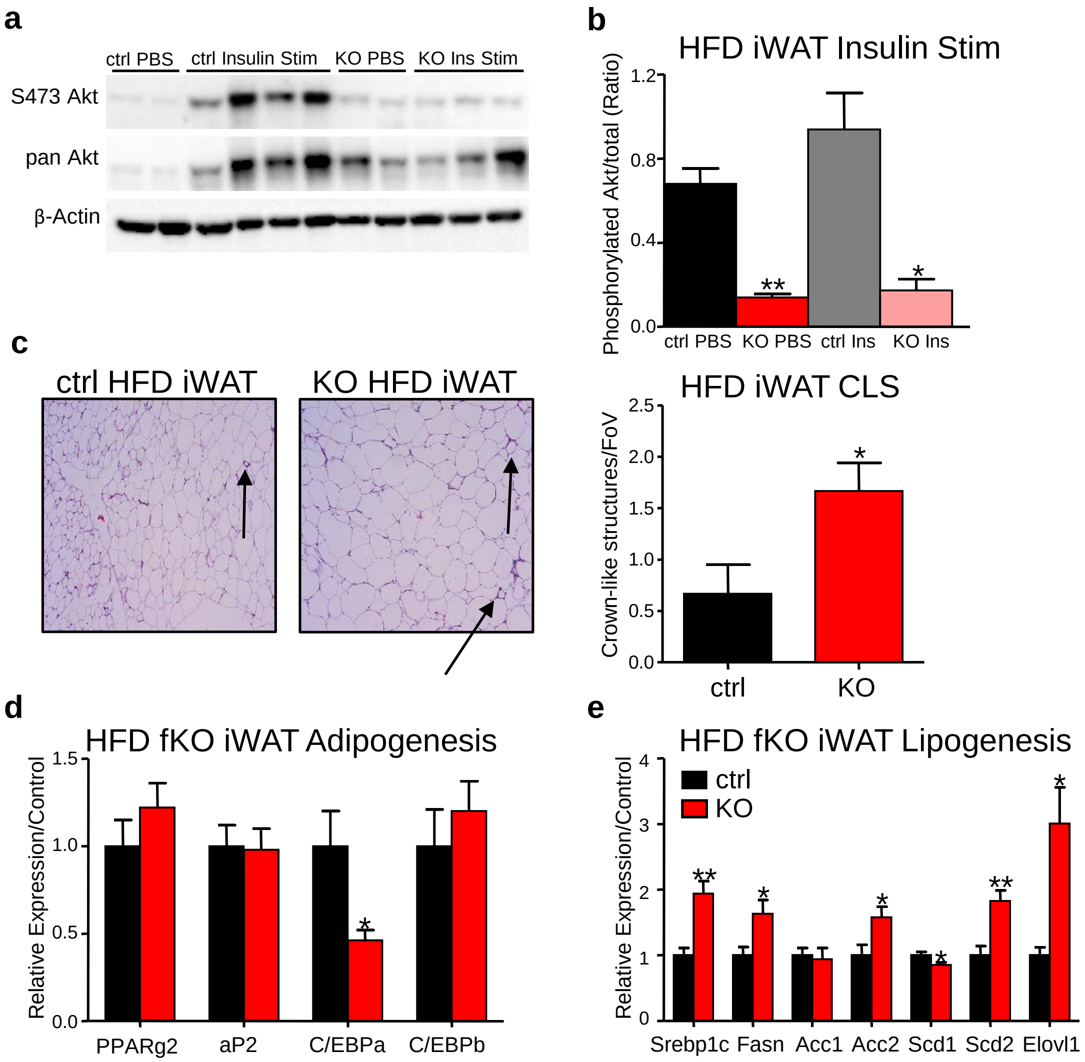

Figure S3. CREB3L3 ablation promotes insulin resistance, crown-like structure formation, and lipogenesis in obese inguinal fat

(**a-b**) Western blot measuring abundance of Akt and Akt phosphorylated at the S473 site in high fat-fed control and fKO iWAT following injection with PBS or insulin and (b) quantification of Western blot results, with abundance of S473 phosphorylation normalized to abundance of total Akt in iWAT (n=2 mice per PBS group; n=3-4 ins-stimulated mice per group).

(**c**) Representative images of H&E-stained iWAT sections following high-fat feeding and quantification of the number of crown-like structures per field of view. Arrows demarcate the presence of crown-like structures (3 images were taken per mouse. n=4-8 mice per group).

(**d-e**) Quantitative PCR for markers of (**d**) adipogenesis or (**e**) lipogenesis in control and fKO iWAT following high-fat feeding (n=5-6 mice per group). Expression was normalized to TBP housekeeping gene and presented as fold change over controls. Data presented as mean +/- SEM with sample sizes listed above. The difference in means was analyzed using Student's t-test where *P<0.05 and **P<0.01.

Figure S4

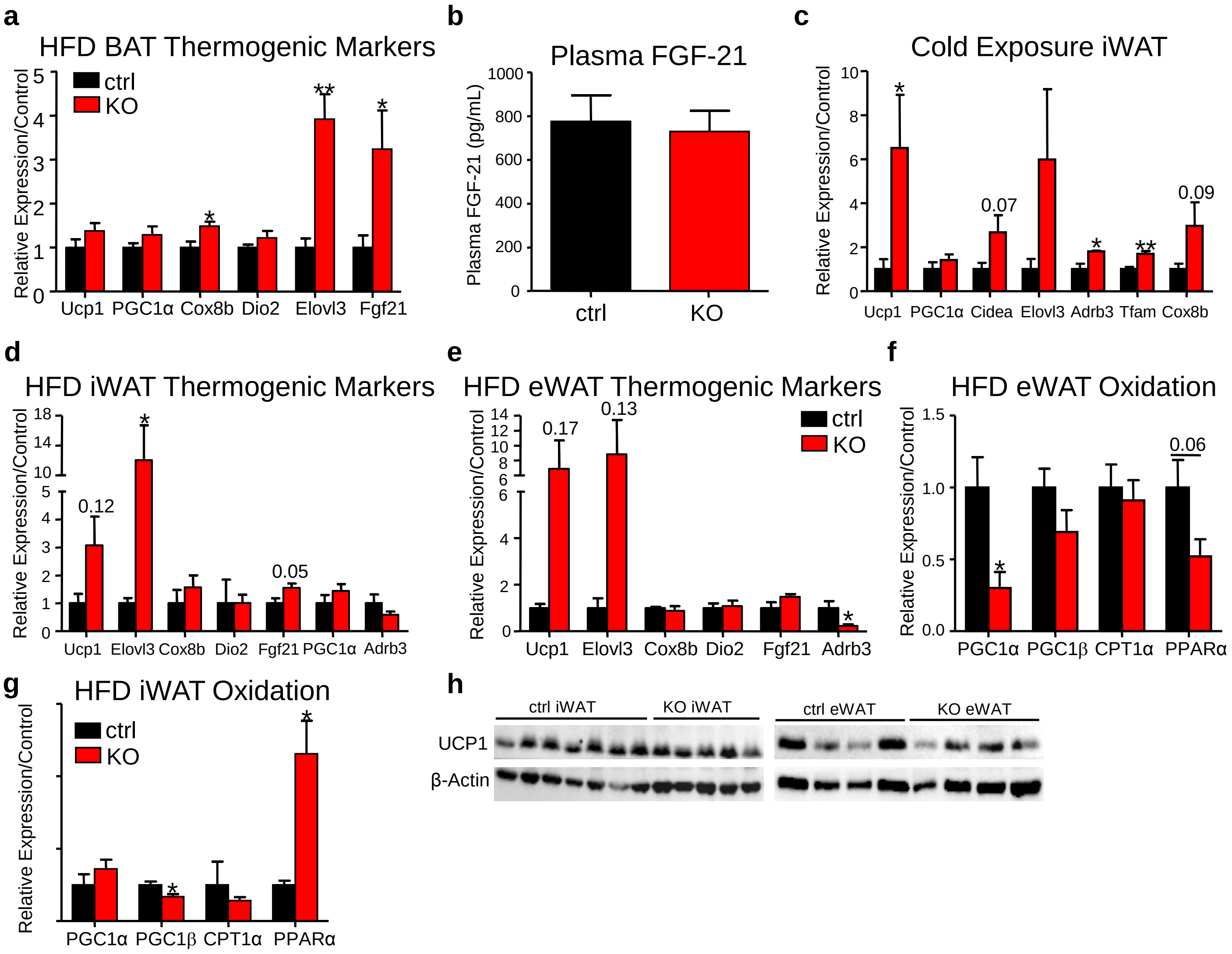

Figure S4. CREB3L3 ablation does not reduce expression of thermogenic markers in obese brown, epididymal, or inguinal fat, nor in cold-exposed inguinal fat

(**a**) Quantitative PCR for markers of thermogenesis and adipocyte browning in brown adipose tissue from control and fKO mice following high-fat feeding (n=5 mice per group). Target gene expression was normalized to TBP and presented as fold change over controls.

(**b**) Quantification of plasma FGF-21 concentration following high-fat feeding using ELISA (n=9 mice per group).

(**c**) Quantitative PCR for markers of thermogenesis and adipocyte browning in inguinal adipose tissue in lean control and fKO mice housed at 6 degrees for 7 days (n=3-4 mice per group).

(**d-e**) Quantitative PCR for markers of thermogenesis and adipocyte browning in (**d**) iWAT or (**e**) eWAT from control and fKO mice following high-fat feeding (n=5-6 mice per group).

(**f-g**) Quantitative PCR for markers of fatty acid oxidation in (**f**) eWAT or (**g**) iWAT from control and fKO mice following high-fat feeding (n=5-6 mice per group).

(**h**) Western blot measuring abundance of UCP1 in iWAT from control and fKO mice following high-fat feeding. Data presented as mean +/- SEM with sample sizes listed above. The difference in means was analyzed using Student's t-test where *$P<0.05$ and **$P<0.01$.

Figure S5

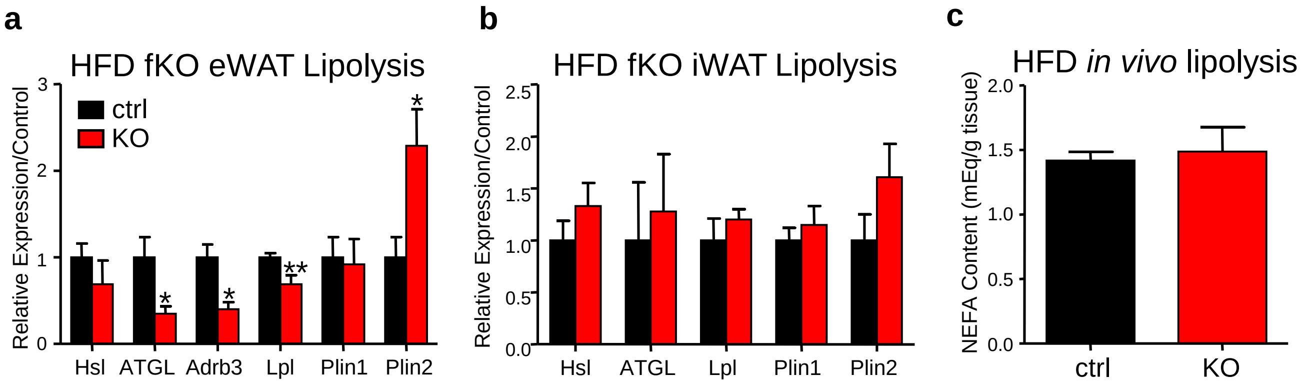

Figure S5. Ablation of CREB3L3 reduces expression of lipolytic markers in obese epidiymal, but not inguinal fat

(**a-b**) Quantitative PCR for markers of lipolysis in (**a**) eWAT or (**b**) iWAT from control and fKO mice following high-fat feeding (n=5 mice per group). Target gene expression was normalized to TBP and presented as fold change over control.

(**c**) Quantification of non-esterified fatty acids (NEFA) from the plasma of high-fat fed control and fKO mice 1h after injection with CL316,243 to induce adipocyte lipolysis (n=5-8 mice per group).

Data presented as mean +/- SEM with sample sizes listed above. The difference in means was analyzed using Student's t-test where *P<0.05 and **P<0.01.

Figure S6

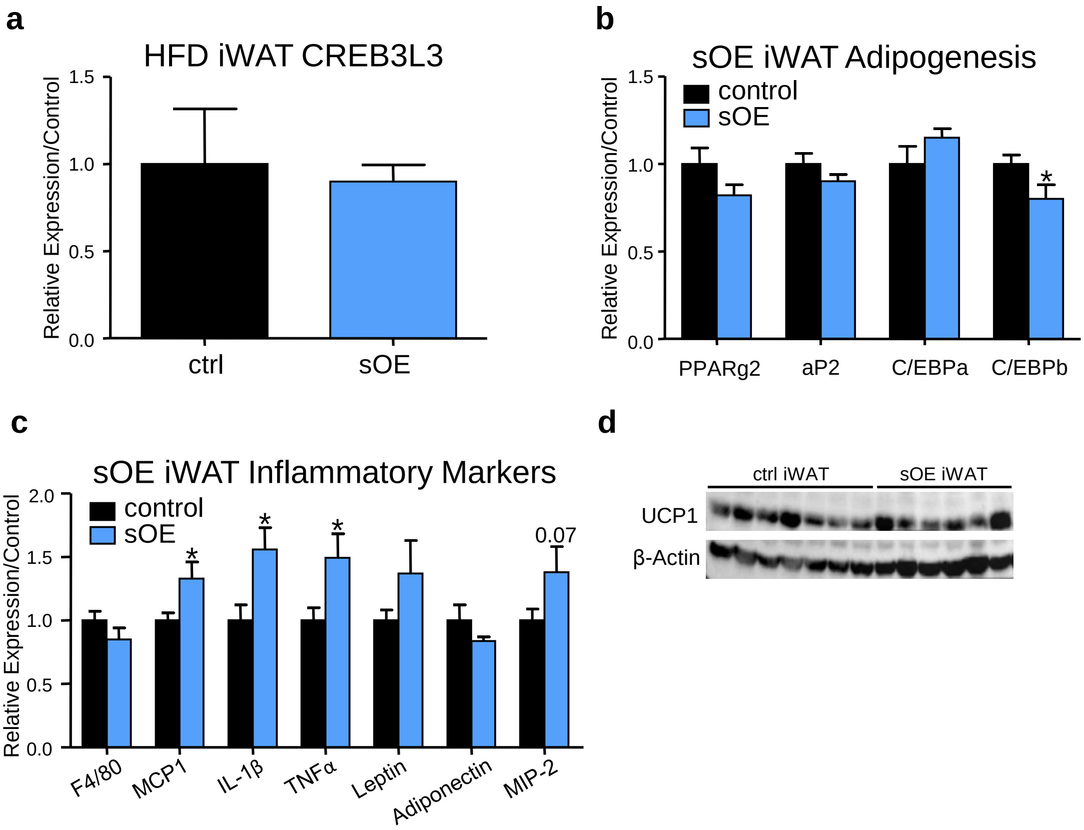

Figure S6. Overexpression of CREB3L3 in inguinal fat increases expression of inflammatory cytokines, but not adipogenic markers or UCP1 protein abundance

(**a**) Quantitative PCR for the expression of CREB3L3 message in iWAT from ctrl and sOE mice following high-fat diet (n=6-8 mice per group). Target gene expression was normalized to TBP and presented as fold change over WT.

(**b-c**) Quantitative PCR for the expression of adipogenic or inflammatory markers in iWAT from ctrl and sOE mice following high-fat diet (n=6-8 mice per group).

(**d**) Western blot measuring abundance of UCP1 in iWAT from ctrl and sOE mice following high-fat feeding. Data presented as mean +/- SEM with sample sizes listed above. The difference in means was analyzed using Student’s t-test where *P<0.05.

Figure S7

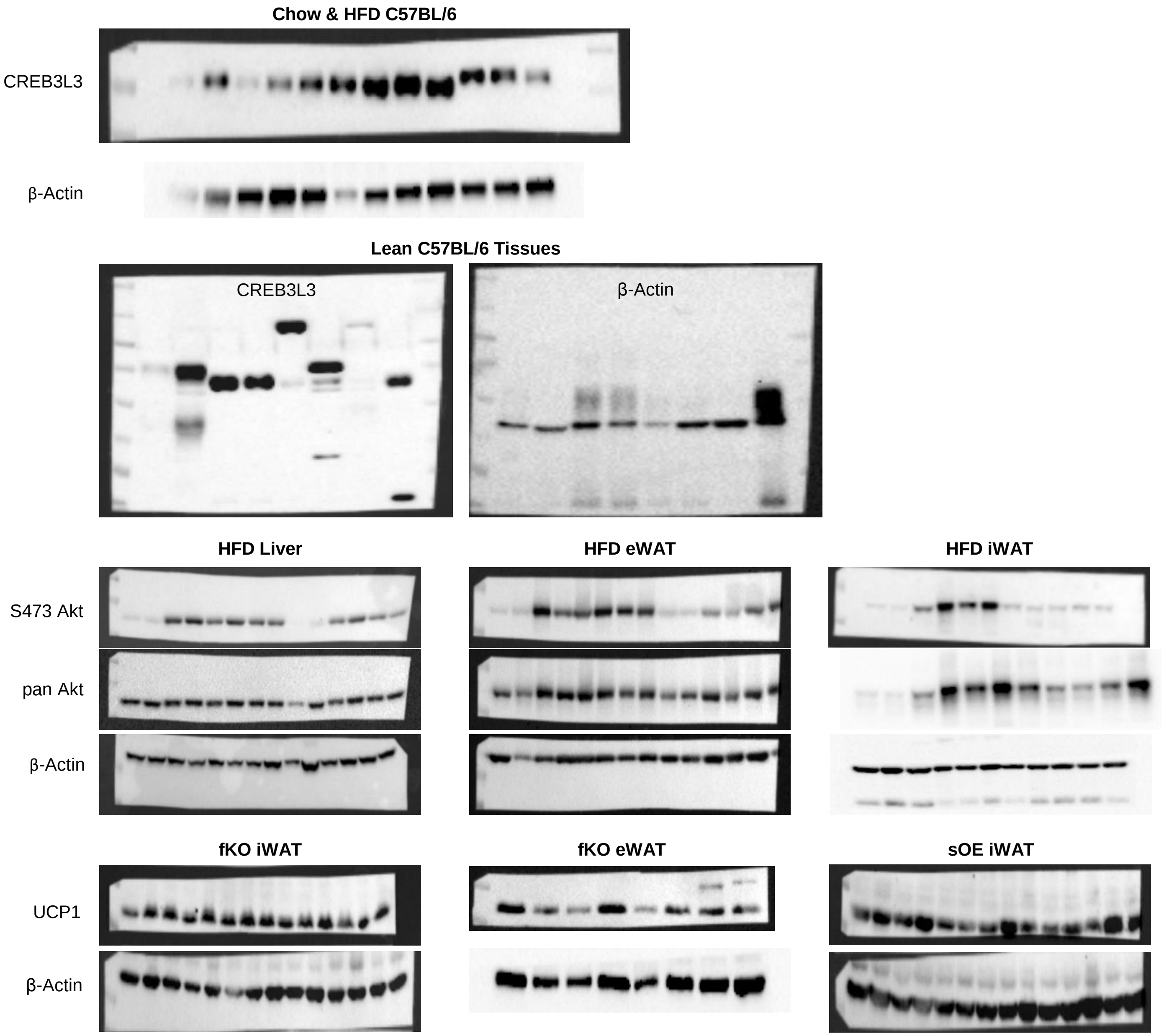

Figure S7. Full length blots for all protein abundance figures

Table S1. Sequence of primers used for qPCR

Gene name	Forward Sequence	Reverse Sequence
CREB3L3	GTGACGCTAGACAGAAACAGTAG	ACCTCCCAAAGATGCTGAAATA
CREB3L3 ORF	GAGAAGAAGCTGCTGGCTAAA	CCGGATCTTTCTGCGGATTT
Igfbp1	GCAGAGTGATGCTGCTTAGA	CTGTGTGAGACGATGAGGAAAT
Pparg1	CTGGCCTCCCTGATGAATAAAG	AGGCTCCATAAAGTCACCAAAG
Pparg2	TCGCTGATGCACTGCCTATG	GAGAGGTCCACAGAGCTGATT
Srebp1c	CCTCTGATCTCATGGCTCATAAC	CTAGGGAACTGTGTGTGTTTCT
Fasn	GCTGCGGAAACTTCAGGAAAT	AGAGACGTGTCACTCCTGGACTT
Fabp4	GATGCCTTTGTGGGAACCTG	CTGTCGTCTGCGGTGATTTC
PGC1a	GACAATCCCGAAGACACTACAG	AGAGAGGAGAGAGAGAGAGAGA
PGC1B	GGTGTTCGGTGAGATTGTAGAG	CTGAACACCGGAAGGTGATAAA
Cox8b	GAACCATGAAGCCAACGACT	GCGAAGTTCACAGTGGTTCC
Cpt1b	TGAGACCAGTCTTAGCCTCTAC	GGCCATTCTTGCAGGAGATAA
Acc1	TGTACAAGCAGTGTGGGCTGGCT	CCACATGGCCTGGCTTGGAGGG
Acc2	GAGGCCGAGAACACAAGAAA	CACCTTCTCTATGACCCTGTTG
Ppara	AAGACTACCTGCTACCGAAATG	AACATTGGGCCGGTTAAGA
Ucp1	CTGCCAGGACAGTACCCAAG	TCAGCTGTTCAAAGCACACA
Elovl3	TCCGCGTTCTCATGTAGGTCT	GGACCTGATGCAACCCTATGA
Cidea	ATCACAACTGGCCTGGTTACG	TACTACCCGGTGTCCATTTCT
Cyc1	GCTACCCATGGTCTCATCGT	CATCATCATTAGGGCCATCC
Tfam	GTCCATAGGCACCGTATTGCG	CCCATGCTGGAAAAACACTTCG
Adrb3	GCTCTGTGTCTCTGGTTAGTTT	GTCCAAGATGGTGCTTAGAGAG
Prdm16	CAGCACGGTGAAGCCATTC	GCGTGCATCCGCTTGTG
F4/80	TTTCCTCGCCTGCTTCTTC	CCCCGTCTCTGTATTCAAC
Ccl2/MCP1	CCACTCACCTGCTGCTACTCAT	TGGTGATCCTCTTGTAGCTCTCC
IL-1b	TGGAGAGTGTGGATCCCAAGCAAT	TGTCCTGACCACTGTTGTTTCCCA
TNF-a	GCCTCTTCTCATTCCTGCTTGT	GGCCATTTGGGAACTTCTCAT
CD74	CCCAGAGAATCTGAAGCATCTTA	CAGGGAGTTCTTGCTCATCTC
Mif	CCAGAACCGCAACTACAGTAAG	GGCAGCGTTCATGTCGTAATA
Resistin	TCAACAAGAAGGAGCTGTGGGACA	ATGGCTTCATCGATGGGACACAGT
Rbp4	ACCTTCTCTAGGTGGACATTAAAC	CATCTTTCAGGGACCTTCAGTAA
Leptin	CCTCATCAAGACCATTGTCACC	TCTCCAGGTCATTGGCTATCTG
Adiponectin	TGTTGCAAGCTCTCCTGTTCCTCT	CATCCAACCTGCACAAGTTCCCTT
Cxcl2/MIP-2	AGTTTGCCTTGACCCTGAAG	TCAGTTAGCCTTGCCTTTGT
Dio2	AAGGCTGCCGAATGTCAACGAATG	TGCTGGTTCAGACTCACCTTGGAA
Fgf21	GCTCTCTATGGATCGCCTCAC	GGTACACATTGTAACCGTCCTC
aP2	CGTCACTTCCACGAGAGTTTAT	TCCCACAGAATGTTGTAGAGTTC
C/EBPα	CTCCCAGAGGACCAATGAAATG	TTAGCCGGAGGAAGCTAAGA
C/EBPβ	CCAAGAAGACGGTGGACAAGC	CAAGTTCCGCAGGGTGCTGA
Plin1	ACTGAAGGGCACCATCTCTA	GGAGGAACTCTACCACCTTCT
Plin2	GGAGGAAAGACTGCCTATTCTG	GTGAGAGGGAAGTACTGGTCTA
Lpl	CCCACAAGTGTAGTCGTCATT	AGGGCTAACATTCCAGCATATC
Scd1	CCCTGCGGATCTTCCTTATC	TGTGTTTCTGAGAACTTGTGGTG
Scd2	CAGTCCCACTCTGACGATAATG	ACAGCTGGGTCCAGTAAGA
